# Comparison of carbon ion and photon reirradiation for recurrent glioblastoma

**DOI:** 10.1007/s00066-021-01844-8

**Published:** 2021-09-14

**Authors:** F. S. Lautenschlaeger, R. Dumke, M. Schymalla, H. Hauswald, B. Carl, M. Stein, U. Keber, A. Jensen, R. Engenhart-Cabillic, F. Eberle

**Affiliations:** 1 Klinik für Strahlentherapie und Radioonkologie, Universitätsklinikum Marburg, Marburg, Germany; 2 Marburg Ion-Beam Therapy Center (MIT), Marburg, Germany; 3 RNS Gemeinschaftspraxis, St. Josefs-Hospital, Wiesbaden, Germany; 4 Klinik für Radio-Onkologie, Universitätsklinikum Heidelberg, Heidelberg, Germany; 5 Klinik für Neurochirurgie, Helios Dr. Horst Schmidt Kliniken Wiesbaden, Wiesbaden, Germany; 6 Klinik für Neurochirurgie, Universitätsklinikum Marburg, Marburg, Germany; 7 Klinik für Neurochirurgie, Universitätsklinikum Gießen, Gießen, Germany; 8 Institut für Neuropathologie, Universitätsklinikum Marburg, Marburg, Germany; 9 Klinik für Strahlentherapie und Radioonkologie, Universitätsklinikum Gießen, Gießen, Germany

**Keywords:** Radiotherapy, Particle therapy, Heavy ion radiotherapy, Glioma, Therapy at recurrence of glioblastoma

## Abstract

**Purpose:**

Purpose of this study was to investigate overall survival in recurrent glioblastoma treated with either carbon ion reirradiation or photon reirradiation.

**Materials and methods:**

In this retrospective study we evaluated 78 consecutive patients with recurrent IDH (Isocitrate dehydrogenase)-wildtype glioblastoma (38 patients carbon ion re-radiotherapy, 40 patients photon re-radiotherapy) treated with either carbon ion reirradiation or stereotactic photon reirradiation. 45 Gy (RBE; 15 fractions) carbon ion reirradiation (CIRT) or 39 Gy (13 fractions) photon reirradiation (FSRT) was administered, respectively. Overall survival was investigated with respect to histological, clinical, and epidemiological features. Kaplan–Meier and multivariate Cox statistics were calculated. A propensity score-matched analysis of the FSRT and CIRT groups using variables from a validated prognosis score was carried out.

**Results:**

The type of reirradiation (CIRT vs. FSRT) significantly influenced overall survival—8.0 months vs. 6.5 months (univariate: *p* = 0.046)—and remained an independent prognostic factor in multivariate analysis (*p* = 0.017). Propensity score-adjusted analysis with CIRT versus FSRT as the dependent variable yielded a significant overall survival advantage for the CIRT group (median OS 8.9 versus 7.2 months, *p* = 0.041, 1‑year survival 29 versus 10%). Adverse events (AE) were evaluated for both subgroups. For the FSRT group no toxicity ≥ grade 4 occurred. For the CIRT subgroup no grade 5 AE occurred, but 1 patient developed a grade 4 radionecrosis. We encountered 4 grade 3 toxicities. One patient developed a zoster at the trunk, 2 progressed in their paresis, and 1 featured progressive dysesthesia.

**Conclusion:**

In conclusion, carbon ion treatment is a safe and feasible treatment option for recurrent glioblastoma. Due to the retrospective nature of the study and two different dose levels for CIRT or FSRT, the improved outcome in CIRT reirradiation might be an effect of higher biological impact from carbon ions or a simple dose-escalation effect. This hypothesis needs prospective testing in larger patient cohorts. A prospective phase III randomized trial is in preparation at our center.

## Introduction

Malignant glioblastoma is the most frequent tumor of the central nervous system (CNS), with an incidence of around 4700 per year in total or 5–8 per 100,000 in Germany [1]. Unfortunately, the current prognosis in glioblastoma remains dismal despite maximum therapy. Current standard of care in newly diagnosed glioblastoma consists of surgery, fractionated chemoradiation with temozolomide up to 60 Gy total dose, and maintenance temozolomide for 6 months [2, 3]. Standard treatment yields a median overall survival (OS) of 14.6 months with progression-free survival (PFS) of only 54% at 6 months and 11% at 24 months.

At recurrence, surgical, chemotherapeutic, and radiotherapeutic options may be available [4]. Tumor control and overall survival by chemotherapy is limited: so far, no chemotherapeutic regimen has shown substantial improvement [4, 5]. Treatment results regarding surgery [4–6] and radiotherapy [7–11] are not undisputed, as these are often based on retrospective analyses. Reirradiation (re-RT) for recurrent glioblastoma has been available for about 20 years [7, 9, 10, 12] and is commonly offered as fractionated stereotactic radiotherapy with photons (FSRT). Some reports on combining FSRT with concurrent chemotherapy at recurrence exist [13–16]. Despite maximum efforts, the outcome in recurrent glioblastoma is bleak, with overall survival rates of only months following treatment of first relapse [7, 9, 10, 12].

Recent developments in radiotherapy technology have led to the availability of new beam qualities, e.g., the possibility of radiotherapy with carbon ions (CIRT) or protons. They feature unique characteristics like high conformality and finite range with the maximal energy deposition at the end of the depth profile, at the so-called Bragg peak. Superior sparing of organs at risk, which is especially important in the case of reirradiation, is possible. Glioblastoma is counted among radioresistant tumors [17]. Due to its different impact on target cells [18], high-LET CIRT offers a possibility to overcome radioresistance [19]. For treatment of primary glioblastoma and high-grade glioma, studies combining photon treatment with a carbon ion boost show promising results regarding overall survival while maintaining a low rate of neurological side effects [20, 21].

Preliminary results for proton or carbon ion re-RT for WHO grade III and IV glioma from other centers are encouraging [22–27]. Favorable overall survival and low toxicity rates have been reported.

In our analysis we compared the overall survival of two cohorts with recurrent glioblastoma, IDH wildtype, treated at our center either with CIRT or FSRT.

## Patients and methods

The patient cohort consisted of 78 patients with recurrent glioblastoma WHO grade IV. 38 patients were treated with CIRT reirradiation and 40 patients were treated with FSRT reirradiation. All patients were diagnosed with glioblastoma, IDH1 and IDH2 wildtype, and no 1p/19q codeletion. Data were collected retrospectively from patient files. According to the standard operating procedures in the clinic, all patients had scheduled clinical visits and MRI scans every third month after radiotherapy. The treatment was conducted between October 2014 and July 2020. Only patients with a complete dataset regarding IDH1/2 mutation status, MGMT promotor methylation, Karnofsky performance index, PTV volume, age, gender, and performance of second surgery were included in the analysis. Data from a part of the previously reported reirradiation high-grade glioma cohort (glioblastoma with complete dataset, *n* = 19) was updated and included in this analysis, too [24].

Treatment at primary diagnosis was conducted for all patients at the University Hospital Giessen and Marburg. After initial diagnosis of the glioblastoma, patients underwent surgery, radiotherapy, and, in most cases, sequential chemotherapy. Conventional or hypofractionated radiotherapy was performed, delivering 40.05–60 Gy (60 Gy median dose). Hypofractionated radiotherapy was administered for elderly patients (age > 70) only.

At recurrence, all cases were discussed in the interdisciplinary neurooncological tumor board.

If feasible, second surgery was performed prior to radiotherapy. For all patients included in this study, reirradiation was performed in a single center. Patients treated with FSRT received a median dose of 39 Gy (range 36 Gy–39 Gy, 95% of prescribed dose covering the PTV) with 3 Gy per fraction. CIRT was conducted with 45 Gy (RBE) with 3 Gy (RBE) per fraction. For each patient, an individual thermoplastic head mask was manufactured. Treatment planning used a CT dataset with a slice thickness of 1.5 mm or 3 mm, registered with a 1-mm T1 3D contrast-enhanced MRI. FSRT planning was conducted using Eclipse V13.5 (Varian Medical Systems, Paolo Alto, USA) planning systems. CIRT treatment plans were calculated with a Siemens Syngo.via (Siemens Healthineers, Erlangen, Germany) treatment planning system. Gross target volumes (GTV) were delineated as contrast enhancement on the T1 3D contrast-enhanced MRI. If a second surgery was performed, the new resection cavity was delineated as the GTV. The clinical target volume (CTV) was defined as 3–5-mm expansion of the GTV, under consideration of anatomical boundaries. Likewise, CTV-to-planning target volume (PTV) expansion was done by expanding the CTV by 3 mm. FSRT plans were stereotactic 3D-CRT plans with 6‑MV photons. CIRT treatment was performed with fixed beams and a raster-scanning technique. Assignment to the different treatment modalities depended only on the availability of the carbon ion facility and the reimbursement of treatment costs by patients’ health insurance, with the largest statutory health insurers usually covering the costs. No selection regarding the medical or histological features was performed. Following the reirradiation, 34 patients received sequential chemotherapy. Sequential chemotherapy at recurrence consisted of temozolomide or lomustine regimes. No concurrent chemotherapy was administered.

Statistical analysis was performed using SPSS v23 (IBM Corp., Armonk, NY, USA). Patient characteristics were checked for statistically significant differences, depending on the level of measurement, either with Mann–Whitney U test or chi^2^ test. Overall survival (OS) from the first day of re-radiotherapy until the last follow-up (censored data) or death was analyzed. For progression-free survival (PFS), time after reirradiation to MRI progression according to RANO, clinical progression, or death was calculated. Variables for OS and PFS included in the analysis were MGMT promotor methylation status, second surgery, and sequential chemotherapy at recurrence, Karnofsky score (KPS), PTV volume, age, sex, and reirradiation with CIRT or FSRT. The variables with ordinal or interval level of measurement were dichotomized. Variables with *p* < 0.1 were included in multivariate analysis. Multivariate analysis used a backward Wald approach with inclusion criteria of 0.1. *P*-values of < 0.05 were considered statistically significant. To achieve balanced basic characteristics, a propensity score-matched analysis with the radiotherapy modality as the dependent variable and prognostic variables known from the modified Combs–Kessel prognostic score as independent variables (patient’s age, time between first radiotherapy and reirradiation, second surgery, KPS, and PTV volume) was conducted [28–31]. A linear regression model with one-to-one nearest neighbor matching and a caliper of 0.2 of the standard deviation of logit of propensity score was used [32]. 26 matched pairs were found, overall survival was calculated using a Kaplan–Meier approach.

Furthermore, patients were sorted into prognosis groups according to the modified Combs score [31]. The crude overall survival for each therapy cohort (CIRT versus FSRT) in each prognosis group (c or d) was calculated and analyzed.

Consent of the local ethics committee was obtained. All patients gave informed consent to the proposed treatment.

## Results

The treatment was completed by 38 patients (20 male, 18 female) in the CIRT group and 40 patients (26 male, 14 female) in the FSRT group. Median age at recurrent disease for the whole collective was 60.8 years (range 19.4–81.4), median age of the CIRT group was 60.6 years (range 19.4–75.7), and median age of the FSRT reirradiation group was 61.3 years (range 36.7–81.4). For 21 patients (52.5%) in the FSRT group and 18 patients (47.4%) in the CIRT group, second surgery had been performed prior to reirradiation. Median PTV at reirradiation for the CIRT group was 89.0 ml (range 13.5–318.0) and median PTV for the FSRT cohort 49.2 ml (range 0.4–301.7). Median PTV for all patients, independent of reirradiation modality, was 64.2 ml. The PTV for retreatment was significantly larger for the CIRT group. A significantly larger number of CIRT patients received temozolomide with primary treatment. Median Karnofsky performance scores were 80% for FSRT re-RT and 70% for CIRT re-RT (range 50–90% for both). Median follow-up was 6.8 months for the FSRT group and 5.8 months for the CIRT group. Patient characteristics are summarized in Table 1.

**Table 1:** Patient characteristics

	Overall	Photons	Carbon-ions	*p* [Δ]
*Patients overall*	78	40	38	–
*Gender*	–	–	–	0.27
Male	46 (59.0%)	26 (65.0%)	20 (52.3%)	–
Female	32 (41.0%)	14 (35.0%)	18 (47.4%)	–
*Age (years)*				
Median	60.8	61.3	60.6	0.46
Range	19.4–81.4	36.7–81.4	19.4–75.7	–
*Temozolomide initial*	70 (89.7%)	32 (80%)	38 (100%)	0.004
*MGMT promotor methylation*	46 (59.0%)	23 (57.5%)	23 (60.5%)	0.79
*Re-resection*	39 (50.0%)	21 (52.5%)	18 (47.4%)	0.65
*PTV volume (ml)*				
Median	64.2	49.2	89.0	0.04
Range	0.4–318.0	0.4–301.7	13.5–318.0	–
*Karnofsky score*				
Median	0.8	0.8	0.7	0.12
Range	50–90%	50–90%	50–90%	–
*Chemotherapy re-challenge*	34 (50.7%)	16 (40.0%)	22 (57.9%)	0.11

*PTV* planing target volume, *MGMT* O6-Methylguanin-DNS-Methyltransferase

### Prognostic factors for OS

**Fig. 1:**
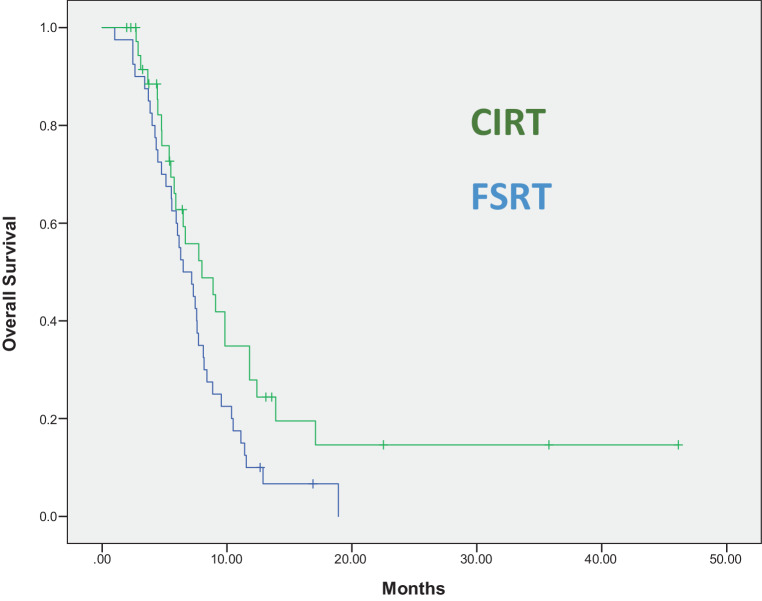
Overall survival after reirradiation with either photons (*blue*) or carbon ions (*green*). Log-rank test revealed a significantly longer median survival for patients treated with carbon ion re-radiotherapy compared to photon reirradiation (*p* = 0.046)

Statistically significant factors in log-rank analysis connected with improved median OS were the choices of CIRT versus FSRT (median OS 8.0 vs. 6.5 months, *p* = 0.046, see Fig. 1). In addition, sequential chemotherapy at recurrence (median OS 7.5 vs. 6.5 months, *p* = 0.044) and conduction of second surgery (median OS 7.7 vs. 6.3 months, *p* = 0.032) were univariate significant prognostic factors for enhanced OS. The 6‑ and 12-month survival rates for CIRT versus FSRT re-radiotherapy were 64 versus 60% and 29 versus 10%, respectively. Variables without a statistically significant influence on OS since MRI recurrence were the patients’ age at recurrence, initial MGMT methylation status, PTV volumes of smaller or larger than 64.2 ml, and the Karnofsky performance score (KPS) at recurrence. *P*-values for log-rank tests for the investigated variables can be found in Table 2.

**Table 2:** Univariate and multivariate *p*-values for log-rank test (overall survival)

	Univariate	Multivariate
Age (split at median of 60.8 years)	0.354	–
Karnofsky score (2 levels, below 80% or over/equal 80%)	0.273	–
Initial methylation of MGMT promotor	0.220	–
Sex (male or female)	0.517	–
Maximum safe resection of recurrent glioblastoma	0.032*	0.037*
Carbon ion vs. photon re-RT	0.046*	0.017*
Chemotherapy re-challenge	0.044*	0.269
PTV size	0.315	–

*P*-values of ≤0.05 were considered statistically significant (*). Univariate: log-rank test, multivariate: cox regression (backward Wald)

Multivariate analyses using cox regression with a backward Wald approach were carried out for all variables with *p*-values ≤ 0.1 in the univariate analysis. The irradiation modality (CIRT vs. FSRT) was a statistically significant parameter for improved overall survival for recurrent glioblastoma in multivariate Cox analysis, with a *p*-value of 0.017. In addition, with a *p*-value of 0.037, the execution of second surgery was a prognostic factor for OS. Repeated chemotherapy as a univariate prognostic factor did not reach significance in multivariate testing (*p* = 0.269).

The modified Combs–Kessel prognosis score was calculated for all cases: 50 patients (24 CIRT and 26 FSRT) were sorted into prognosis group c, 23 patients (13 CIRT and 10 FSRT) were ranked into the worst prognosis group d.

For the patients in group c, median overall survival with CIRT was 8.9 months versus 6.2 months for FSRT (*p* = 0.046). The patients in prognosis group d featured a median overall survival of 7.7 months for CIRT and 4.3 months for FSRT (*p* = 0.075).

**Fig. 2:**
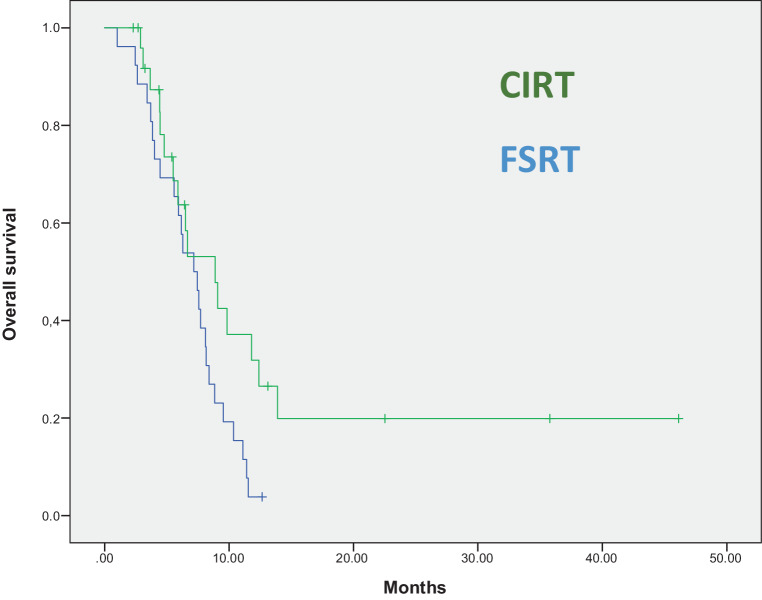
Propensity score-matched analysis with the variables from the modified Combs score as matching parameters. Reirradiation with carbon ions leads to statistically significant longer overall survival (*p* = 0.041)

Propensity score matching to balance for possible confounders was conducted. As the dependent variable, the reirradiation technique (CIRT versus FSRT) was selected. As independent variables the prognostic variables from the Combs–Kessel score were selected. 26 matched pairs were derived by this technique. Kaplan–Meier analysis and log-rank test featured a superior median survival for carbon ion-treated patients (8.9 months vs. 7.2 months, *p* = 0.041; Fig. 2 and Table 2).

### Progression-free survival

Univariate analysis of the whole collective and of the matched-pair group showed a trend towards better progression-free survival for the patients treated with CIRT (5.5 months vs. 3.9 months, *p* = 0.063; for the propensity score-matched group 5.7 months vs. 3.9 months, *p* = 0.085). However statistical significance was not reached. The other univariate factors tested (KPS, age, MGMT status, PTV size, sequential chemotherapy after re-RT, second surgery) did not qualify for multivariate analysis (*p* > 0.1 in univariate analysis).

### Adverse events

Grade 5 toxicities according to CTCAE 4.0 occurred in none of the investigated cases. No toxicity > CTCAE grade III were documented for the FSRT cohort. Therapy-related adverse events >grade II according CTCAE v5.0 were assessed for patients treated with carbon ions. We encountered 4 grade 3 toxicities and one grade 4 toxicity within the CIRT cohort. One CIRT patient developed grade 4 radionecrosis and required surgical intervention. One patient developed a zoster at the trunk, 2 progressed in their paresis, and 1 featured progressive dysesthesia. All patients with the described adverse effects were treated with a prescription dose of 45 Gy RBE ^12^C-ions. The PTV volumes were in the range between 25 ml and 224 ml. Patient age ranged from 48 years to 74 years. The time from primary radiotherapy to CIRT was between 7 and 15 months.

## Discussion

For patients with glioblastoma, recurrence of disease is frequent. Despite this fact, no standard pattern of care has yet been established for recurrent glioblastoma. Reirradiation with modern techniques is reported to be safe and feasible and can be offered as a therapeutic option for recurrent glioblastoma. In this study we investigated the impact of CIRT reirradiation in the treatment of recurrent glioblastoma. We analyzed patients treated consecutively between October 2014 and June 2020 with either CIRT or FSRT reirradiation. Only patients with glioblastoma, IDH wildtype, were included in the analysis. Due to better dosimetric sparing of organs at risk in CIRT, it was possible for a larger median reirradiation PTV to be treated. For the FSRT reirradiation cohort, a median OS of 6.5 months, a 6-month survival rate of 60%, and a 1-year survival rate of 10% was calculated. The patients treated with CIRT at recurrence reached a median OS of 8.0 months. A 6-month OS of 64% and 1‑year OS of 29% was achieved. Furthermore, our propensity score-matched analysis featured a statistically better overall survival for the CIRT cohort (8.9 months median OS for carbon ion and 7.2 months median OS for FSRT). Both carbon ion cohorts, the unmatched as well as the propensity score-matched cohort, performed slightly better with respect to overall survival compared to the FSRT patients from our database. None of the patients in our CIRT treatment group had grade 5 toxicities. However, 1 patient (3.7%) developed grade 4 radionecrosis and 4 patients (14.8%) with grade 3 adverse events required medical intervention. This rate of grade 3 toxicities was slightly higher compared to other retrospective studies but in line with results from prospective photon studies [33].

To our knowledge, full publications of particle beam re-RT for recurrent glioma are scarce (2 × proton re-RT and 1 × carbon ion re-RT with more than 10 patients and 1 paper with only 5 patients included have been identified). Furthermore, a few studies were published as abstract only. Galle et al. reported on 13 patients with recurrent glioblastoma treated with protons with a median dose of 54 Gy RBE. Median PTV at re-RT was 84.4 ml and all patients underwent a second surgery. A median OS of 8.2 months after reirradiation was achieved while maintaining a favorable toxicity profile. No acute toxicities > CTCAE grade II were reported [26]. Another report on proton beam re-RT of recurrent glioma grade III and IV was published by Scartoni et al., in which 33 patients were reirradiated with 36-Gy RBE protons. The median overall survival was determined to be 8.7 months while the 6‑ and 12-month survival rates were 100 and 33%, respectively [25]. The third publication on proton re-RT by Mizumoto et al. included data from 5 glioblastoma patients. However, due to the limited number of patients, the data of Mizumoto had to be interpretated rather like a collection of case reports than as an analysis of a patient cohort [27].

Results from the Cinderella trial on reirradiation of recurrent high-grade glioma with 45 Gy RBE carbon ions has not been made available as a full publication yet. Preliminary results from 41 patients with WHO grade III–IV glioma were reported as an ASCO abstract. For WHO grade III and IV glioma, a median overall survival after reirradiation of 10.5 months (322 days) was reported [23]. Unfortunately, no subgroup analysis for WHO grade III and IV tumors is available. The authors report no therapy-related toxicity ≥grade 3. At DEGRO’s 2020 meeting, Adeberg et al. reported on their analysis of patients with WHO grade III and WHO grade IV glioma from the Cinderella cohort. The authors performed a 1:1 matched-pair analysis including 59 patients treated with 45 Gy RBE CIRT and 59 patients treated by mildly hypofractionated FSRT with 39 Gy. Re-RT with carbon ions led to a favorable OS of 13.1 months compared to 8.8 months. One toxicity grade III according to CTCAE v4.03 and no grade 4 or 5 toxicities occurred [22]. Again, no subgroup analysis for WHO grade IV tumors was available. In addition, Eberle et al. published data on 23 patients with relapsed glioblastoma treated with 45 Gy RBE CIRT reirradiation. A median overall survival of 12 months after MRI diagnosis of recurrence was achieved. Toxicity levels were low, no grade 4 or 5 toxicities occurred [24].

Particle beam re-radiotherapy and especially CIRT re-RT allows for safe application of high re-RT doses (i.e., 45 Gy [RBE] carbon ions, 15 fractions, EQD2 = 48.8 Gy, α/β = 10 Gy; EQD2 = 56.3 Gy, α/β = 2 Gy) and safe irradiation of large target volumes.

Taking our data and the data published by Adeberg et al. for a matched-pair or propensity score-matched cohort into account, CIRT might outperform mildly hypofractionated FSRT re-RT approaches with respect to an enhanced overall survival of patients [22]. The reason for this might be an enhanced biological efficacy of carbon ions in hypoxic and necrotic tumors, as indicated by in vitro studies [19]. However, due to the mismatch of EQD2 between CIRT and FSRT doses, a simple dose-escalation effect cannot finally be excluded on the basis of the data by Adeberg et al. or the data from this study.

For both treatment modalities (CIRT versus FSRT) in our study, the median OS as well as the 6‑month and 12-month OS fitted into the range of values reported in literature [5, 7, 9, 10, 31, 33]. However, the definition of the time period which had been taken into account for OS calculation is heterogenous amongst the photon re-RT publications. Some authors used the date of recurrence diagnosed by MRI, while others calculated from the date of the first session of re-RT. Taking the rather short OS times of months into account, small differences in the measured time due to different starting points might play a statistically significant role.

Numerous publications on reirradiation with photons exist. A recent meta-analysis by Kazmi et al. included 33 studies on EBRT of glioblastoma recurrences. The irradiation concepts were very heterogeneous among the studies. Kazmi et al. reported a used dose range between 23 and 50 Gy, EQD2, α/β = 10 Gy. A 6-month overall survival of 73% and a 12-month overall survival of 36% are reported summarized across all studies. The median age of patients included in this meta-analysis is 53 years [33]. Our results for FSRT-treated patients showed worse overall survival rates of 60% at 6 months and 10% at 1 year. However, the median age of our patients was 61.3 years and the median PTV size was larger than reported in the meta-analysis and other studies. Both factors are important determinants of overall survival according to the validated prognosis scores [31, 34–36]. Thus, the shorter survival times in our collective might result from an overall prognostically worse patient collective.

For a collective of reirradiated patients, Kessel et al. reported on the development of their modified Combs score. They reported a median overall survival of 8.1 months for patients sorted into group c and 5.5 months for patients included in the worst prognosis group d [31]. For our patient collective, subgroup analysis of prognosis group c and prognosis group d (according to the modified Combs score) yielded significantly better overall survival for patients of group c irradiated with carbon ions (8.9 versus 6.2 months; 2.7 months enhanced OS for CIRT re-RT, *p* = 0.046) and showed a trend towards better median overall survival for the CIRT reirradiation cohort for prognosis group d. Our FSRT reirradiation patients sorted into group d reached a median overall survival of 4.3 months, while our carbon ion reirradiation cohort featured a median overall survival of 7.7 months. Both CIRT re-RT subgroups performed better than the corresponding photon comparators and better than the photon prognosis subgroups published by Kessel et al. [31].

In our analysis sequential chemotherapy was not a significant prognostic factor for OS or PFS in multivariate analysis. In the literature, different chemotherapeutic agents like temozolomide, lomustine, or bevacizumab (in combination with lomustine) have been evaluated [16]. So far, no phase III trial featuring an advantage regarding the OS of patients with recurrent glioblastoma when treated with chemotherapy exists [37]. Concurrent chemotherapy with reirradiation was investigated in a couple of analyses [13–15, 38]. A retrospective, secondary analysis of the RTOG 0525 trial led to a 2-month enhanced OS for combined radiochemotherapy compared to chemotherapy alone [38]. Highly selected patients with small recurrences (median PTV of 12.1 to 32.1 cm^3^) were investigated by trials using hypofractionated FSRT with photons up to doses from 20 Gy (ED 10 Gy) to 25 Gy (ED 5 Gy). The reported toxicity levels were low [13–15]. Median overall survival and 6‑ and 12-month survival were in the range reported in the literature for trials without concurrent chemotherapy. However, for small tumor volumes, concurrent application of chemotherapy and reirradiation seemed to be possible without excessive risk. Concurrent chemotherapy with CIRT of glioma has not been evaluated so far. With data featuring low toxicity for hypofractionated CIRT as well as for concurrent chemotherapy with hypofractionated FSRT available, the simultaneous combination of chemotherapy and CIRT might be feasible for future trials.

## Conclusion

Radiotherapy with carbon ions for recurrent glioblastoma is a safe and effective treatment. Patients might benefit from an enhanced median overall survival when comparing our CIRT and FSRT re-RT collectives. The level of adverse events is low and in line with data reported in the literature for photon re-RT or the few reports on particle beam re-RT. Of course, the CIRT and FSRT reirradiation concepts used different radiation doses (45 Gy RBE versus 39 Gy), so a simple dose-related effect cannot be disproved with the data at hand. Therefore, to conclusively evaluate the effects of carbon ion re-radiotherapy, prospective studies comparing photon and carbon ion treatment approaches at recurrence are warranted. We are planning to conduct a randomized multicenter study comparing stereotactic photon reirradiation with carbon ion reirradiation at the same dose level.
